# Draft Genome Sequence of *Caedibacter varicaedens*, a Kappa Killer Endosymbiont Bacterium of the Ciliate *Paramecium biaurelia*

**DOI:** 10.1128/genomeA.01310-15

**Published:** 2015-11-05

**Authors:** Haruo Suzuki, Amy L. Dapper, Craig E. Jackson, Heewook Lee, Vikas Pejaver, Thomas G. Doak, Michael Lynch, John R. Preer

**Affiliations:** a Department of Biology, Indiana University, Bloomington, Indiana, USA; b Institute for Advanced Biosciences, Keio University, Fujisawa, Kanagawa, Japan; c Department of Computer Science and Informatics, School of Informatics and Computing, Indiana University, Bloomington, Indiana, USA; d School of Public and Environmental Affairs, Indiana University, Bloomington, Indiana, USA

## Abstract

*Caedibacter varicaedens* is a kappa killer endosymbiont bacterium of the ciliate *Paramecium biaurelia*. Here, we present the draft genome sequence of *C. varicaedens*.

## GENOME ANNOUNCEMENT

The genus *Caedibacter* comprises species “*Candidatus* Caedibacter acanthamoebae” (endosymbiont of acanthamoebae), *Caedibacter caryophilus* (*Paramecium caudatum* symbiont), *Caedibacter macronucleorum* (*Paramecium duboscqui* symbiont), *Caedibacter paraconjugatus* (*Paramecium biaurelia* symbiont), *Caedibacter pseudomutans* (*Paramecium tetraurelia* symbiont), *Caedibacter taeniospiralis* (*Paramecium caudatum* symbiont), and *Caedibacter varicaedens* (*Paramecium biaurelia* symbiont) (1). Of the *Caedibacter* species described to date, only the draft genome sequence of “*Ca.* Caedibacter acanthamoebae” has been determined (2). *Caedibacter varicaedens* is a kappa killer endosymbiont bacterium of the ciliate *Paramecium biaurelia* (3). To study the evolution of the *Caedibacter*-*Paramecium* symbiosis, we determined the draft genome sequence of *C. varicaedens*.

Whole-genome sequencing was performed using the Roche 454 pyrosequencing platform at the Center for Genomics and Bioinformatics (CGB), Indiana University. The sequencing reads were assembled using Newbler version 2.5.3. After removal of possible contaminant sequences, i.e., contigs with unusual values of statistics such as %GC content and coverage (sequencing depth), the resulting assembly contains 142 contigs consisting of 1,686,852 bp, with a G+C content of 42.1%. Genome annotation was performed using Prokka v1.11 (4), yielding 41 tRNA genes, 1 copy of a 16S-23S-5S rRNA gene, and 1,726 protein-coding sequences (CDS), of which 588 (34%) are hypothetical proteins and 8 contain a protein motif (Pfam PF11747.2) related to the killing trait deposited in Pfam: the protein families database (5). SignalP 4.1 (6) predicted 86 signal peptides. The genome sequence was analyzed using G-language Genome Analysis Environment version 1.9.0 (7), available at http://www.g-language.org.

We performed similarity searches of the 1,726 protein sequences against the UniRef90 sequence databases (8) using BLASTP (9) with the E value cutoff of 1e-20 and assigned the most similar (best hit) protein sequence information. Of the 1,726 proteins, 1,222 (71%) had matches with 1,090 unique records in the UniRef90 database, of which 100 (8%) are transposase family proteins. The species distribution of the BLAST best hits in the UniRef90 database showed that 565 (46%) of the 1,222 hits had top matches with sequences from “*Ca.* Caedibacter acanthamoebae,” followed by “*Candidatus* Paracaedibacter symbiosus” with 36 (2.9%) best BLAST hits. Both of the species, as well as *C. varicaedens*, belong to the order *Rickettsiales* of *Alphaproteobacteria* (1). The genome sequence data in this study will provide useful information for understanding the evolution of symbiosis between *C. varicaedens* and the host *P. biaurelia*.

### Nucleotide sequence accession numbers.

The sequence has been deposited as a whole-genome shotgun project at DDBJ/EMBL/GenBank under the accession number BBVC00000000. The version described in this paper is the first version, BBVC01000000 (BBVC01000001 to BBVC01000142).
